# Wait Times for Psychiatric Specialist Services in Australia

**DOI:** 10.1001/jamanetworkopen.2024.61947

**Published:** 2025-02-26

**Authors:** Ou Yang, Yuting Zhang

**Affiliations:** 1 Melbourne Institute: Applied Economic & Social Research, University of Melbourne, Melbourne, Victoria, Australia

## Abstract

This cross-sectional study examines the differences in wait times for general psychiatric specialist consultations in Australia from 2011 to 2022, stratified by location, socioeconomic status, and type of consultation.

## Introduction

One substantial challenge within Australia’s universal health care system is increasing specialist service wait times, which vary by location and socioeconomic status.^1,2^ Psychiatry has become one of Australia’s most consulted outpatient specialties and is heavily subsidized by Medicare.^3^ While some studies have examined mental health service wait times for children and adolescents in Australia,^4,5^ no study, to our knowledge, has analyzed national data. Our study aims to fill this gap.

## Methods

Patients in Australia generally require a general practitioner (GP) referral to a medical specialist, including psychiatric specialists. We analyzed national outpatient wait times from GP referral to first psychiatric specialist appointment using Australia’s whole-of-population Medicare Benefit Schedule (MBS) data from 2011 to 2022 accessed via the Person Level Integrated Data Asset (PLIDA).^6^ We reported wait times for in-person and telehealth services. We also studied differences in wait times between residents from major cities vs regional and remote areas. All MBS items related to psychiatric specialist in-person and telehealth services were selected, grouped, and assessed accordingly (Supplement 1). The Office of Research Ethics and Integrity at the University of Melbourne approved this cross-sectional study and waived informed consent because deidentified data were used. We followed the STROBE reporting guideline. Data were analyzed from April 4 to September 24, 2024, using Stata, version 18.0 (StataCorp LLC)

## Results

A total of 1 459 366 observations were included in the sample. The Table shows the trend over time in the mean wait times for in-person psychiatric specialist services in Australia. The increase since 2020 was large vs the years before 2019. Compared with 2011 when the median (IQR) wait time was 15 (1-52) days, wait time rose to 50 (10-113) days in 2022, with similar upward trends across the 25th, 75th, and 95th percentiles.

**Table.** Wait Time From Referral to First Appointment With a Psychiatrist Stratified by In-Person or Telehealth Services From 2011 to 2022^a^

Year	Time, d					No. of observations
	Mean	Median (IQR)	25th percentile	75th percentile	95th percentile	
**In-person appointments**						
2011	51	15 (1-52)	1	52	226	165 139
2012	49	15 (1-50)	1	50	213	134 283
2013	49	18 (2-52)	2	52	203	127 533
2014	49	19 (2-53)	2	53	204	124 366
2015	48	20 (3-53)	3	53	203	122 179
2016	50	22 (4-58)	4	58	206	117 913
2017	51	23 (5-61)	5	61	210	115 950
2018	53	26 (6-64)	6	64	214	115 534
2019	52	28 (6-66)	6	66	197	111 907
2020	57	30 (7-72)	7	72	214	95 770
2021	66	39 (8-90)	8	90	234	93 703
2022	77	50 (10-113)	10	113	258	76 687
**Telehealth services**						
2020	60	32 (8-73)	8	73	233	12 860
2021	88	57 (15-125)	15	125	288	18 015
2022	68	41 (11-95)	11	95	235	27 527

^a^ Medicare Benefit Schedule data from 2011 to 2022 obtained through the Person Level Integrated Data Asset were used.^6^

Telehealth services had minimal use prepandemic, with only 2066 observations recorded between 2011 and 2019 vs more than 10 000 annually since 2020. The Table reports telehealth summary statistics from 2020 onward, showing the median (IQR) wait time of 32 (8-73) days in 2020 rising significantly to 57 (15-125) days in 2021 before decreasing to 41 (11-95) days in 2022. In comparison, in-person services had median (IQR) wait times of 30 (7-72) days, 39 (8-90) days, and 50 (10-113) days in 2020, 2021, and 2022, respectively.

Furthermore, there was considerable variation in wait times for in-person and telehealth services. In 2022, in-person wait times ranged from 10 days at the 25th percentile to 258 days at the 95th percentile, while telehealth wait times ranged from 11 to 235 days. This wide variation persisted over time. For both service types, the medians are lower than means, indicating a skewed distribution with a long right tail.

The Figure shows geographical disparities in mean wait times for psychiatric services based on patients’ locations. Regional and remote areas consistently experienced longer waits than major cities for both services. This disparity persisted except for in-person services during the pandemic’s early phase when regional and remote areas had fewer lockdowns and less stringent movement restrictions compared with major cities. The difference was most pronounced for telehealth in 2021.

**Figure.**
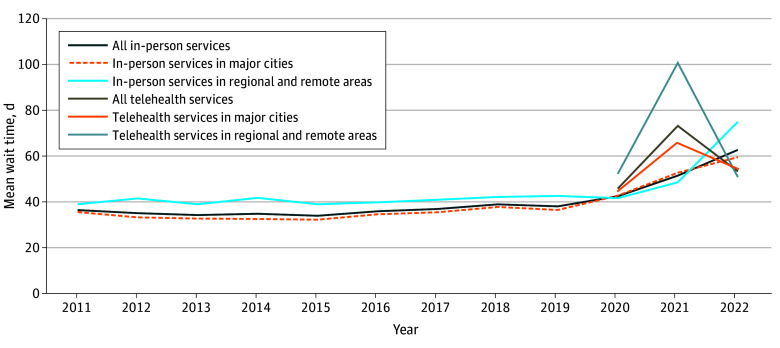
Wait Time From Referral to First Appointment With a Psychiatrist Stratified by In-Person or Telehealth Services and by Areas in Australia

## Discussion

We found a general temporal trend of increasing wait time for psychiatric services across Australia, with a disproportionately higher burden in regional and remote areas. Wider adoption of telehealth services did not reduce this disparity. The increasing wait time underscores the need for continued attention to health care access and distribution of psychiatric services across Australia. Study limitations include a focus on wait times without identifying reasons for increased times. We also did not stratify trends by demographic variables. Future research should examine wait time differences across age groups to identify at-risk subpopulations and factors in increasing wait time for psychiatric services.

## Notes

Supplement 1. **eMethods.** MBS Item Codes Analyzed in the Study

Supplement 2.
Data Sharing Statement
