# Supplementary material for: Wait Times for Psychiatric Specialist Services in Australia
Source: JAMA Netw Open. 2025 Feb 26;8(2):e2461947. doi: 10.1001/jamanetworkopen.2024.61947 (PMC11866023; doi:10.1001/jamanetworkopen.2024.61947)
Supplement: Supplement 1. — eMethods. MBS Item Codes Analyzed in the Study [file jamanetwopen-e2461947-s001.pdf]

Supplementary Online Content

Yang O, Zhang Y. Waiting times for psychiatric specialist services in Australia. *JAMA Netw Open*. 2025;8(2):e2461947. doi:10.1001/jamanetworkopen.2024.61947

eMethods. MBS Item Codes Analyzed in the Study

This supplemental material has been provided by the authors to give readers additional information about their work.

© 2025 Yang O et al. *JAMA Network Open*.

MBS Item Codes Analyzed in the Study

The Australian MBS items related to psychiatric specialist in-person attendances analysed include: 288, 289, 291, 293, 294, 296, 297, 299, 300, 302, 304, 306, 308, 310, 312, 314, 316, 318, 319, 320, 322, 324, 326, 328, 330, 332, 334, 336, 338, 342, 344, 346, 348, 350, 352, 361, 364, 366, 367, 369 and 370.

The Australian MBS items related to psychiatric specialist telehealth attendances analysed include: 353, 355 – 359, 91827 – 91831, 92434 – 92437, 92455 – 92466.

Item Description:

288: Professional attendance on a patient by a consultant physician practising in the consultant physician's specialty of psychiatry if: (a) the attendance is by video conference; and (b) item 291, 293, 296, 300, 302, 304, 306, 308, 310, 312, 314, 316, 318, 319, 348, 350 or 352 applies to the attendance; and (c) the patient is not an admitted patient; and (d) the patient: (i) is located both: (A) within a telehealth eligible area; and (B) at the time of the attendance-at least 15 kms by road from the physician; or (ii) is a care recipient in a residential care service; or (iii) is a patient of: (A) an Aboriginal Medical Service; or (B) an Aboriginal Community Controlled Health Service; for which a direction made under subsection 19(2) of the Act applies

289: Professional attendance of at least 45 minutes in duration at consulting rooms or hospital, by a consultant physician in the practice of the consultant physician's specialty of psychiatry, following referral of the patient to the consultant by a referring practitioner, for assessment, diagnosis and preparation of a treatment and management plan for a patient under 13 years with autism or another pervasive developmental disorder, if the consultant psychiatrist does all of the following: (a) undertakes a comprehensive assessment and makes a diagnosis (if appropriate, using information provided by an eligible allied health provider); (b) develops a treatment and management plan which must include the following: (i) an assessment and diagnosis of the patient's condition; (ii) a risk assessment; (iii) treatment options and decisions; (iv) if necessary-medication recommendations; (c) provides a copy of the treatment and management plan to the referring practitioner; (d) provides a copy of the treatment and management plan to one or more allied health providers, if appropriate, for the treatment of the patient; (other than attendance on a patient for whom payment has previously been made under this item or item 135, 137 or 139)

291: Professional attendance of more than 45 minutes in duration at consulting rooms by a consultant physician in the practice of the consultant physician's specialty of psychiatry, if: (a) the attendance follows referral of the patient to the consultant for an assessment or management by a medical practitioner in general practice (including a general practitioner, but not a specialist or consultant physician) or a participating nurse practitioner; and (b) during the attendance, the consultant: (i) uses an outcome tool (if clinically appropriate); and (ii) carries out a mental state examination; and (iii) makes a psychiatric diagnosis; and (c) the consultant decides that it is clinically appropriate for the patient to be managed by the referring practitioner without ongoing treatment by the consultant; and (d) within 2 weeks after the attendance, the consultant: (i) prepares a written diagnosis of the patient; and (ii) prepares a written management plan for the patient that: (A) covers the next 12 months; and (B) is appropriate to the patient's diagnosis; and (C) comprehensively evaluates the patient's biological,

© 2025 Yang O et al. *JAMA Network Open*.

psychological and social issues; and (D) addresses the patient's diagnostic psychiatric issues; and (E) makes management recommendations addressing the patient's biological, psychological and social issues; and (iii) gives the referring practitioner a copy of the diagnosis and the management plan; and (iv) if clinically appropriate, explains the diagnosis and management plan, and a gives a copy, to: (A) the patient; and (B) the patient's carer (if any), if the patient agrees

293: Professional attendance of more than 30 minutes but not more than 45 minutes in duration at consulting rooms by a consultant physician in the practice of the consultant physician's specialty of psychiatry, if: (a) the patient is being managed by a medical practitioner or a participating nurse practitioner in accordance with a management plan prepared by the consultant in accordance with item 291; and (b) the attendance follows referral of the patient to the consultant for review of the management plan by the medical practitioner or a participating nurse practitioner managing the patient; and (c) during the attendance, the consultant: (i) uses an outcome tool (if clinically appropriate); and (ii) carries out a mental state examination; and (iii) makes a psychiatric diagnosis; and (iv) reviews the management plan; and (d) within 2 weeks after the attendance, the consultant: (i) prepares a written diagnosis of the patient; and (ii) revises the management plan; and (iii) gives the referring practitioner a copy of the diagnosis and the revised management plan; and (iv) if clinically appropriate, explains the diagnosis and the revised management plan, and gives a copy, to: (A) the patient; and (B) the patient's carer (if any), if the patient agrees; and (e) in the preceding 12 months, a service to which item 291 applies has been provided; and (f) in the preceding 12 months, a service to which this item applies has not been provided

296: Professional attendance of more than 45 minutes in duration by a consultant physician in the practice of the consultant physician's speciality of psychiatry following referral of the patient to him or her by a referring practitioner-an attendance at consulting rooms if the patient: (a) is a new patient for this consultant psychiatrist; or (b) has not received a professional attendance from this consultant psychiatrist in the preceding 24 months; other than attendance on a patient in relation to whom this item, or item 297 or 299 or any of items 300 to 308, has applied in the preceding 24 months

297: Professional attendance of more than 45 minutes by a consultant physician in the practice of the consultant physician's speciality of psychiatry following referral of the patient to him or her by a referring practitioner-an attendance at hospital if the patient: (a) is a new patient for this consultant psychiatrist; or (b) has not received a professional attendance from this consultant psychiatrist in the preceding 24 months; other than attendance on a patient in relation to whom this item, or item 296 or 299 or any of items 300 to 308, has applied in the preceding 24 months (H)

299: Professional attendance of more than 45 minutes by a consultant physician in the practice of the consultant physician's speciality of psychiatry following referral of the patient to him or her by a referring practitioner-an attendance at a place other than consulting rooms or a hospital if the patient: (a) is a new patient for this consultant psychiatrist; or (b) has not received a professional attendance from this consultant psychiatrist in the preceding 24 months; other than attendance on a patient in relation to whom this item, or item 296 or 297 or any of items 300 to 308, has applied in the preceding 24 months

300: Professional attendance by a consultant physician in the practice of the consultant physician's specialty of psychiatry following referral of the patient to him or her by a referring practitioner-an attendance of not more than 15 minutes in duration at consulting rooms, if that attendance and another attendance to which item 296 or any of items 300 to 308 applies have not exceeded 50 attendances in a calendar year for the patient

302: Professional attendance by a consultant physician in the practice of the consultant physician's specialty of psychiatry following referral of the patient to him or her by a referring practitioner-an attendance of more than 15 minutes, but not more than 30 minutes, in duration at consulting rooms, if that attendance and another attendance to which item 296 or any of items 300 to 308 applies have not exceeded 50 attendances in a calendar year for the patient

304: Professional attendance by a consultant physician in the practice of the consultant physician's specialty of psychiatry following referral of the patient to him or her by a referring practitioner-an attendance of more than 30 minutes, but not more than 45 minutes, in duration at consulting rooms), if that attendance and another attendance to which item 296 or any of items 300 to 308 applies have not exceeded 50 attendances in a calendar year for the patient

306: Professional attendance by a consultant physician in the practice of the consultant physician's specialty of psychiatry following referral of the patient to him or her by a referring practitioner-an attendance of more than 45 minutes, but not more than 75 minutes, in duration at consulting rooms, if that attendance and another attendance to which item 296 or any of items 300 to 308 applies have not exceeded 50 attendances in a calendar year for the patient

308: Professional attendance by a consultant physician in the practice of the consultant physician's specialty of psychiatry following referral of the patient to him or her by a referring practitioner-an attendance of more than 75 minutes in duration at consulting rooms), if that attendance and another attendance to which item 296 or any of items 300 to 308 applies have not exceeded 50 attendances in a calendar year for the patient

310: Professional attendance by a consultant physician in the practice of the consultant physician's specialty of psychiatry following referral of the patient to the consultant physician by a referring practitioner-an attendance of not more than 15 minutes in duration at consulting rooms, if that attendance and another attendance to which item 296 or any of items 300 to 308 applies exceed 50 attendances in a calendar year for the patient

312: Professional attendance by a consultant physician in the practice of the consultant physician's specialty of psychiatry following referral of the patient to the consultant physician by a referring practitioner-an attendance of more than 15 minutes, but not more than 30 minutes, in duration at consulting rooms, if that attendance and another attendance to which item 296 or any of items 300 to 308 applies exceed 50 attendances in a calendar year for the patient

314: Professional attendance by a consultant physician in the practice of the consultant physician's specialty of psychiatry following referral of the patient to the consultant physician by a referring practitioner-an attendance of more than 30 minutes, but not more than 45 minutes, in duration at consulting rooms, if that attendance and another attendance to which item 296 or any of items 300 to 308 applies exceed 50 attendances in a calendar year for the patient

316: Professional attendance by a consultant physician in the practice of the consultant physician's specialty of psychiatry following referral of the patient to the consultant physician

© 2025 Yang O et al. *JAMA Network Open*.

by a referring practitioner-an attendance of more than 45 minutes, but not more than 75 minutes, in duration at consulting rooms, if that attendance and another attendance to which item 296 or any of items 300 to 308 applies exceed 50 attendances in a calendar year for the patient

318: Professional attendance by a consultant physician in the practice of the consultant physician's specialty of psychiatry following referral of the patient to the consultant physician by a referring practitioner-an attendance of more than 75 minutes in duration at consulting rooms, if that attendance and another attendance to which item 296 or any of items 300 to 308 applies exceed 50 attendances in a calendar year for the patient

319: Professional attendance by a consultant physician in the practice of the consultant physician's specialty of psychiatry following referral of the patient to the consultant physician by a referring practitioner'—an attendance of more than 45 minutes in duration at consulting rooms, if the patient has: (a) been diagnosed as suffering severe personality disorder, anorexia nervosa, bulimia nervosa, dysthymic disorder, substance-related disorder, somatoform disorder or a pervasive development disorder; and (b) for patients 18 years and over'—been rated with a level of functional impairment within the range 1 to 50 according to the Global Assessment of Functioning Scale; if that attendance and another attendance to which item 296 or any of items 300 to 308 applies have not exceeded 160 attendances in a calendar year for the patient

320: Professional attendance by a consultant physician in the practice of the consultant physician's specialty of psychiatry following referral of the patient to the consultant physician by a referring practitioner-an attendance of not more than 15 minutes in duration at hospital

This supplementary material has been provided by the authors to give readers additional information about their work.

322: Professional attendance by a consultant physician in the practice of the consultant physician's specialty of psychiatry following referral of the patient to the consultant physician by a referring practitioner-an attendance of more than 15 minutes, but not more than 30 minutes, in duration at hospital

324: Professional attendance by a consultant physician in the practice of the consultant physician's specialty of psychiatry following referral of the patient to the consultant physician by a referring practitioner-an attendance of more than 30 minutes, but not more than 45 minutes, in duration at hospital

326: Professional attendance by a consultant physician in the practice of the consultant physician's specialty of psychiatry following referral of the patient to the consultant physician by a referring practitioner-an attendance of more than 45 minutes, but not more than 75 minutes, in duration at hospital

328: Professional attendance by a consultant physician in the practice of the consultant physician's specialty of psychiatry following referral of the patient to the consultant physician by a referring practitioner-an attendance of more than 75 minutes in duration at hospital

330: Professional attendance by a consultant physician in the practice of the consultant physician's specialty of psychiatry following referral of the patient to the consultant physician by a referring practitioner-an attendance of not more than 15 minutes in duration if that attendance is at a place other than consulting rooms or hospital

© 2025 Yang O et al. *JAMA Network Open*.

332: Professional attendance by a consultant physician in the practice of the consultant physician's specialty of psychiatry following referral of the patient to the consultant physician by a referring practitioner-an attendance of more than 15 minutes, but not more than 30 minutes, in duration if that attendance is at a place other than consulting rooms or hospital

334: Professional attendance by a consultant physician in the practice of the consultant physician's specialty of psychiatry following referral of the patient to the consultant physician by a referring practitioner-an attendance of more than 30 minutes, but not more than 45 minutes, in duration if that attendance is at a place other than consulting rooms or hospital

336: Professional attendance by a consultant physician in the practice of the consultant physician's specialty of psychiatry following referral of the patient to the consultant physician by a referring practitioner-an attendance of more than 45 minutes, but not more than 75 minutes, in duration if that attendance is at a place other than consulting rooms or hospital

338: Professional attendance by a consultant physician in the practice of the consultant physician's specialty of psychiatry following referral of the patient to the consultant physician by a referring practitioner-an attendance of more than 75 minutes in duration if that attendance is at a place other than consulting rooms or hospital

342: Group psychotherapy (including any associated consultations with a patient taking place on the same occasion and relating to the condition for which group therapy is conducted) of not less than 1 hour in duration given under the continuous direct supervision of a consultant physician in the practice of the consultant physician's specialty of psychiatry, involving a group of 2 to 9 unrelated patients or a family group of more than 3 patients, each of whom is referred to the consultant physician by a referring practitioner-each patient

344: Group psychotherapy (including any associated consultations with a patient taking place on the same occasion and relating to the condition for which group therapy is conducted) of not less than 1 hour in duration given under the continuous direct supervision of a consultant physician in the practice of the consultant physician's specialty of psychiatry, involving a family group of 3 patients, each of whom is referred to the consultant physician by a referring practitioner-each patient

346: Group psychotherapy (including any associated consultations with a patient taking place on the same occasion and relating to the condition for which group therapy is conducted) of not less than 1 hour in duration given under the continuous direct supervision of a consultant physician in the practice of the consultant physician's specialty of psychiatry, involving a family group of 2 patients, each of whom is referred to the consultant physician by a referring practitioner-each patient

348: Professional attendance by a consultant physician in the practice of the consultant physician's specialty of psychiatry, following referral of the patient to the consultant physician by a referring practitioner, involving an interview of a person other than the patient of not less than 20 minutes, but less than 45 minutes, in duration, in the course of initial diagnostic evaluation of a patient

350: Professional attendance by a consultant physician in the practice of the consultant physician's specialty of psychiatry, following referral of the patient to the consultant physician

© 2025 Yang O et al. *JAMA Network Open*.

by a referring practitioner, involving an interview of a person other than the patient of not less than 45 minutes in duration, in the course of initial diagnostic evaluation of a patient

352: Professional attendance by a consultant physician in the practice of the consultant physician's specialty of psychiatry, following referral of the patient to the consultant physician by a referring practitioner, involving an interview of a person other than the patient of not less than 20 minutes in duration, in the course of continuing management of a patient-if that attendance and another attendance to which this item applies have not exceeded 4 in a calendar year for the patient

361: Professional attendance by a consultant physician in the practice of the consultant physician's specialty of psychiatry following referral of the patient to the consultant physician by a referring practitioner-a telepsychiatry consultation of more than 45 minutes in duration, if the patient: (a) either: (i) is a new patient for this consultant physician; or (ii) has not received a professional attendance from this consultant physician in the preceding 24 months; and (b) is located in a regional, rural or remote area; other than attendance on a patient in relation to whom this item, item 296, 297 or 299, or any of items 300 to 346 and 353 to 370, has applied in the preceding 24 month period

364: Professional attendance by a consultant physician in the practice of the consultant physician's specialty of psychiatry following referral of the patient to the consultant physician by a referring practitioner-a face-to-face consultation of not more than 15 minutes in duration, if: (a) the patient has had a telepsychiatry consultation to which any of items 353 to 358 and 361 applies before that attendance; and (b) that attendance and another attendance to which any of items 296 to 308, 353 to 358 and 361 to 370 applies have not exceeded 50 attendances in a calendar year for the patient

366: Professional attendance by a consultant physician in the practice of the consultant physician's specialty of psychiatry following referral of the patient to the consultant physician by a referring practitioner-a face-to-face consultation of more than 15 minutes, but not more than 30 minutes, in duration, if: (a) the patient has had a telepsychiatry consultation to which any of items 353 to 358 and 361 applies before that attendance; and (b) that attendance and another attendance to which any of items 296 to 308, 353 to 358 and 361 to 370 applies have not exceeded 50 attendances in a calendar year for the patient

367: Professional attendance by a consultant physician in the practice of the consultant physician's specialty of psychiatry following referral of the patient to the consultant physician by a referring practitioner-a face-to-face consultation of more than 30 minutes, but not more than 45 minutes, in duration, if: (a) the patient has had a telepsychiatry consultation to which any of items 353 to 358 and 361 applies before that attendance; and (b) that attendance and another attendance to which any of items 296 to 308, 353 to 358 and 361 to 370 applies have not exceeded 50 attendances in a calendar year for the patient

369: Professional attendance by a consultant physician in the practice of the consultant physician's specialty of psychiatry following referral of the patient to the consultant physician by a referring practitioner-a face-to-face consultation of more than 45 minutes, but not more than 75 minutes, in duration, if: (a) the patient has had a telepsychiatry consultation to which any of items 353 to 358 and 361 applies before that attendance; and (b) that attendance and

another attendance to which any of items 296 to 308, 353 to 358 and 361 to 370 applies have not exceeded 50 attendances in a calendar year for the patient

370: Professional attendance by a consultant physician in the practice of the consultant physician's specialty of psychiatry following referral of the patient to the consultant physician by a referring practitioner-a face-to-face consultation of more than 75 minutes in duration, if: (a) the patient has had a telepsychiatry consultation to which any of items 353 to 358 and 361 applies before that attendance; and (b) that attendance and another attendance to which any of items 296 to 308, 353 to 358 and 361 to 370 applies have not exceeded 50 attendances in a calendar year for the patient

353: Professional attendance by a consultant physician in the practice of the consultant physician's specialty of psychiatry following referral of the patient to the consultant physician by a referring practitioner-a telepsychiatry consultation of not more than 15 minutes in duration, if: (a) that attendance and another attendance to which any of items 353 to 358 and 361 applies have not exceeded 12 attendances in a calendar year for the patient; and (b) that attendance and another attendance to which any of items 296 to 308, 353 to 358 and 361 to 370 applies have not exceeded 50 attendances in a calendar year for the patient

355: Professional attendance by a consultant physician in the practice of the consultant physician's specialty of psychiatry following referral of the patient to the consultant physician by a referring practitioner-a telepsychiatry consultation of more than 15 minutes, but not more than 30 minutes, in duration, if: (a) that attendance and another attendance to which any of items 353 to 358 and 361 applies have not exceeded 12 attendances in a calendar year for the patient; and (b) that attendance and another attendance to which any of items 296 to 308, 353 to 358 and 361 to 370 applies have not exceeded 50 attendances in a calendar year for the patient

356: Professional attendance by a consultant physician in the practice of the consultant physician's specialty of psychiatry following referral of the patient to the consultant physician by a referring practitioner-a telepsychiatry consultation of more than 30 minutes, but not more than 45 minutes, in duration, if: (a) that attendance and another attendance to which any of items 353 to 358 and 361 applies have not exceeded 12 attendances in a calendar year for the patient; and (b) that attendance and another attendance to which any of items 296 to 308, 353 to 358 and 361 to 370 applies have not exceeded 50 attendances in a calendar year for the patient

357: Professional attendance by a consultant physician in the practice of the consultant physician's specialty of psychiatry following referral of the patient to the consultant physician by a referring practitioner-a telepsychiatry consultation of more than 45 minutes, but not more than 75 minutes, in duration, if: (a) that attendance and another attendance to which any of items 353 to 358 and 361 applies have not exceeded 12 attendances in a calendar year for the patient; and (b) that attendance and another attendance to which any of items 296 to 308, 353 to 358 and 361 to 370 applies have not exceeded 50 attendances in a calendar year for the patient

358: Professional attendance by a consultant physician in the practice of the consultant physician's specialty of psychiatry following referral of the patient to the consultant physician by a referring practitioner-a telepsychiatry consultation of more than 75 minutes in duration, if:

© 2025 Yang O et al. *JAMA Network Open*.

(a) that attendance and another attendance to which any of items 353 to 358 and 361 applies have not exceeded 12 attendances in a calendar year for the patient; and (b) that attendance and another attendance to which any of items 296 to 308, 353 to 358 and 361 to 370 applies have not exceeded 50 attendances in a calendar year for the patient

359: Professional attendance by a consultant physician in the practice of the consultant physician's specialty of psychiatry-a telepsychiatry consultation of more than 30 minutes but not more than 45 minutes in duration, if: (a) the patient is being managed by a medical practitioner or a participating nurse practitioner in accordance with a management plan prepared by the consultant physician in accordance with item 291; and (b) the attendance follows referral of the patient to the consultant physician for review of the management plan by the referring practitioner managing the patient; and (c) during the attendance, the consultant physician: (i) uses an outcome tool (if clinically appropriate); and (ii) carries out a mental state examination; and (iii) makes a psychiatric diagnosis; and (iv) reviews the management plan; and (d) within 2 weeks after the attendance, the consultant physician: (i) prepares a written diagnosis of the patient; and (ii) revises the management plan; and (iii) gives the referring practitioner a copy of the diagnosis and the revised management plan; and (iv) if clinically appropriate, explains the diagnosis and the revised management plan, and gives a copy, to: (A) the patient; and (B) the patient's carer (if any), if the patient agrees; and (e) the patient is located in a regional, rural or remote area; and (f) in the preceding 12 months, a service to which item 291 applies has been performed; and (g) in the preceding 12 months, a service to which this item or item 293 applies has not been performed

91827: Telehealth attendance for a person by a consultant psychiatrist; if: (a) the attendance follows a referral of the patient to the consultant psychiatrist by a referring practitioner; and (b) the attendance was not more than 15 minutes duration.

91828: Telehealth attendance for a person by a consultant psychiatrist; if: (a) the attendance follows a referral of the patient to the consultant psychiatrist by a referring practitioner; and (b) the attendance was at least 15 minutes, but not more than 30 minutes in duration.

91829: Telehealth attendance for a person by a consultant psychiatrist; if: (a) the attendance follows a referral of the patient to the consultant psychiatrist by a referring practitioner; and (b) the attendance was at least 30 minutes, but not more than 45 minutes in duration.

91830: Telehealth attendance for a person by a consultant psychiatrist; if: (a) the attendance follows a referral of the patient to the consultant psychiatrist by a referring practitioner; and (b) the attendance was at least 45 minutes, but not more than 75 minutes in duration.

91831: Telehealth attendance for a person by a consultant psychiatrist; if: (a) the attendance follows a referral of the patient to the consultant psychiatrist by a referring practitioner; and (b) the attendance was at least 75 minutes in duration.

92434: Telehealth attendance of at least 45 minutes in duration, by a consultant physician in the practice of the consultant physician's specialty of psychiatry, following referral of the patient to the consultant physician by a referring practitioner, for assessment, diagnosis and preparation of a treatment and management plan for a patient under 13 years with autism or another pervasive developmental disorder, if the consultant physician does all of the following: (a) undertakes a comprehensive assessment and makes a diagnosis (if appropriate, using information provided by an eligible allied health provider); (b) develops a treatment and

management plan which must include the following: (i) an assessment and diagnosis of the patient's condition; (ii) a risk assessment; (iii) treatment options and decisions; (iv) if necessary'—medication recommendations; (c) provides a copy of the treatment and management plan to the referring practitioner; (d) provides a copy of the treatment and management plan to one or more allied health providers, if appropriate, for the treatment of the patient; (other than attendance on a patient for whom payment has previously been made under this item, or item 135, 137, 139, 289 of the general medical services table, or item 92140, 92141, 92142 or 92145)

92435: Telehealth attendance of more than 45 minutes in by a consultant physician in the practice of the consultant physician's specialty of psychiatry, if: (a) the attendance follows referral of the patient to the consultant for an assessment or management by a medical practitioner in general practice (not including a specialist or consultant physician) or a participating nurse practitioner; and (b) during the attendance, the consultant: (i) uses an outcome tool (if clinically appropriate); and (ii) carries out a mental state examination; and (iii) makes a psychiatric diagnosis; and (c) the consultant decides that it is clinically appropriate for the patient to be managed by the referring practitioner without ongoing treatment by the consultant; and (d) within 2 weeks after the attendance, the consultant: (i) prepares a written diagnosis of the patient; and (ii) prepares a written management plan for the patient that: (A) covers the next 12 months; and (B) is appropriate to the patient's diagnosis; and (C) comprehensively evaluates the patient's biological, psychological and social issues; and (D) addresses the patient's diagnostic psychiatric issues; and (E) makes management recommendations addressing the patient's biological, psychological and social issues; and (iii) gives the referring practitioner a copy of the diagnosis and the management plan; and (iv) if clinically appropriate, explains the diagnosis and management plan, and a gives a copy, to: (A) the patient; and (B) the patient's carer (if any), if the patient agrees; and (e) in the preceding 12 months, a service to which this item or item 291 of the general medical services table applies has not been provided

92436: Telehealth attendance of more than 30 minutes but not more than 45 minutes in duration by a consultant physician in the practice of the consultant physician's specialty of psychiatry, if: (a) the patient is being managed by a medical practitioner or a participating nurse practitioner in accordance with a management plan prepared by the consultant in accordance with item 291 or 92435; and (b) the attendance follows referral of the patient to the consultant for review of the management plan by the medical practitioner or a participating nurse practitioner managing the patient; and (c) during the attendance, the consultant: (i) uses an outcome tool (if clinically appropriate); and (ii) carries out a mental state examination; and (iii) makes a psychiatric diagnosis; and (iv) reviews the management plan; and (d) within 2 weeks after the attendance, the consultant: (i) prepares a written diagnosis of the patient; and (ii) revises the management plan; and (iii) gives the referring practitioner a copy of the diagnosis and the revised management plan; and (iv) if clinically appropriate, explains the diagnosis and the revised management plan, and gives a copy, to: (A) the patient; and (B) the patient's carer (if any), if the patient agrees; and (e) in the preceding 12 months, a service to which item 291 of the general medical services table or92435 applies has been provided; and (f) in the preceding 12 months, a service to which this item, or item 293 of the general medical services table applies has not been provided

© 2025 Yang O et al. *JAMA Network Open*.

92437: Telehealth attendance of more than 45 minutes in duration by a consultant physician in the practice of the consultant physician's speciality of psychiatry following referral of the patient to the consultant physician by a referring practitioner: (a) if the patient: (i) is a new patient for this consultant physician; or (ii) has not received an attendance from this consultant physician in the preceding 24 months; and (b) the patient has not received an attendance under this item, or item 91827 to 91831, 91837 to 91839, 92455 to 92457, or item296, 297, 299 or300 to 346 of the general medical services table, in the preceding 24 months

92455: Telehealth attendance for group psychotherapy (including any associated consultations with a patient taking place on the same occasion and relating to the condition for which group therapy is conducted): (a) of not less than 1 hour in duration; and (b) given under the continuous direct supervision of a consultant physician in the practice of the consultant physician's specialty of psychiatry; and (c) involving a group of 2 to 9 unrelated patients or a family group of more than 3 patients, each of whom is referred to the consultant physician by a referring practitioner; '—each patient

92456: Telehealth attendance for group psychotherapy (including any associated consultations with a patient taking place on the same occasion and relating to the condition for which group therapy is conducted): (a) of not less than 1 hour in duration; and (b) given under the continuous direct supervision of a consultant physician in the practice of the consultant physician's specialty of psychiatry; and (c) involving a family group of 3 patients, each of whom is referred to the consultant physician by a referring practitioner; '—each patient

92457: Telehealth attendance for group psychotherapy (including any associated consultations with a patient taking place on the same occasion and relating to the condition for which group therapy is conducted): (a) of not less than 1 hour in duration; and (b) given under the continuous direct supervision of a consultant physician in the practice of the consultant physician's specialty of psychiatry; and (c) involving a family group of 2 patients, each of whom is referred to the consultant physician by a referring practitioner; '—each patient

92458: Telehealth attendance by a consultant physician in the practice of the consultant physician's specialty of psychiatry, following referral of the patient to the consultant physician by a referring practitioner, involving an interview of a person other than the patient of not less than 20 minutes, but less than 45 minutes, in duration, in the course of initial diagnostic evaluation of a patient. .

92459: Telehealth attendance by a consultant physician in the practice of the consultant physician's specialty of psychiatry, following referral of the patient to the consultant physician by a referring practitioner, involving an interview of a person other than the patient of not less than 45 minutes in duration, in the course of initial diagnostic evaluation of a patient.

92460: Telehealth attendance by a consultant physician in the practice of the consultant physician's specialty of psychiatry, following referral of the patient to the consultant physician by a referring practitioner, involving an interview of a person other than the patient of not less than 20 minutes in duration, in the course of continuing management of a patient'—if that attendance and another attendance to which this item or item 352 of the general medical services table applies have not exceeded 4 in a calendar year for the patient

92461: Telehealth attendance for a person by a consultant psychiatrist; if: (a)the attendance follows a referral of the patient to the consultant psychiatrist by a referring practitioner; and

(b)the attendance was not more than 15 minutes duration; and (c) the patient to whom the service is provided is admitted to hospital

92462: Telehealth attendance for a person by a consultant psychiatrist; if: (a)the attendance follows a referral of the patient to the consultant psychiatrist by a referring practitioner; and (b)the attendance was at least 15 minutes, but not more than 30 minutes in duration; and (c) the patient to whom the service is provided is admitted to hospital

92463: Telehealth attendance for a person by a consultant psychiatrist; if: (a)the attendance follows a referral of the patient to the consultant psychiatrist by a referring practitioner; and (b)the attendance was at least 30 minutes, but not more than 45 minutes in duration; and (c) the patient to whom the service is provided is admitted to hospital

92464: Telehealth attendance for a person by a consultant psychiatrist; if: (a)the attendance follows a referral of the patient to the consultant psychiatrist by a referring practitioner; and (b)the attendance was at least 45 minutes, but not more than 75 minutes in duration; and (c) the patient to whom the service is provided is admitted to hospital

92465: Telehealth attendance for a person by a consultant psychiatrist; if: (a)the attendance follows a referral of the patient to the consultant psychiatrist by a referring practitioner; and (b)the attendance was at least 75 minutes in duration; and (c) the patient to whom the service is provided is admitted to hospital

92466: Telehealth attendance of more than 45 minutes in duration by a consultant physician in the practice of the consultant physician's speciality of psychiatry following referral of the patient to the consultant physician by a referring practitioner, if the patient to whom the service is provided: (a) either: (i) is a new patient for this consultant physician; or (ii) has not received an attendance from this consultant physician in the preceding 24 months; and (b) is admitted to hospital

© 2025 Yang O et al. *JAMA Network Open*.
