# Supplementary material for: Wait Times for Psychiatric Specialist Services in Australia
Source: JAMA Netw Open. 2025 Feb 26;8(2):e2461947. doi: 10.1001/jamanetworkopen.2024.61947 (PMC11866023; doi:10.1001/jamanetworkopen.2024.61947)
Supplement: Supplement 2. — Data Sharing Statement [file jamanetwopen-e2461947-s002.pdf]

Data Sharing Statement

Yang. Wait Times for Psychiatric Specialist Services in Australia. *JAMA Netw Open*. Published February 26, 2025. doi:10.1001/jamanetworkopen.2024.61947

Data
Data available: No

Additional Information
Explanation for why data not available: We do not own these data. Researchers can request data through the Australian Bureau of Statistics.
